# Fate of Dissolved Organic Matter and Cooperation Behavior of Coagulation: Fenton Combined with MBR Treatment for Pharmaceutical Tail Water

**DOI:** 10.3390/molecules30122520

**Published:** 2025-06-09

**Authors:** Jian Wang, Chunxiao Zhao, Feng Qian, Jie Su, Hongjie Gao

**Affiliations:** 1 State Key Laboratory of Environmental Criteria and Risk Assessment, Chinese Research Academy of Environmental Sciences, Beijing 100012, China; wangjian0092024@163.com (J.W.); 15101122950@163.com (C.Z.); gaohj@craes.org.cn (H.G.); 2 Key Laboratory of Estuarine and Coastal Environment of the Ministry of Ecology and Environment, Chinese Research Academy of Environmental Sciences, Beijing 100012, China; 3 Institute of Water Eco-Environment Research, Chinese Research Academy of Environmental Sciences, Beijing 100012, China

**Keywords:** pharmaceutical tail water, fenton, coagulation, MBR

## Abstract

In this study, the treatment of pharmaceutical tail water (PTW) by coagulation, Fenton combined with membrane bioreactor (MBR), was studied. Optimal parameters were obtained according to batch experiment and central composite design (CCD). Results showed that Polymeric Ferric Sulfate (PFS) was the best coagulant for original pharmaceutical tailwater due to less dosage and higher removal efficiency to TOC, COD, NH_4_^+^-N and UV_254m_, with the optimized pH = 7.25 and 0.53 g/L PFS dosage. The best coagulation performance was achieved when the mixer was stirred at 250 rpm for 3 min, 60 rpm for 10 min, and then left to stand for 60 min. Coagulation mainly removed organics with molecular weight above 10 kDa. After treated by coagulation, 43.1% TOC removal efficiency of PTW was obtained by Fenton reaction with 11.6 mmol/L H_2_O_2_, 3.0 mmol/L FeSO_4_, pH = 3.3 and T = 50 min. A type of common macromolecule aromatic amino acid compounds which located Ex = 250 nm and Em = 500 nm was the main reason that caused the high TOC concentration in the effluent. Stable COD and NH_4_^+^-N removal efficiencies in the MBR reactor within 10 d were observed when the mixture of pre-treated PTW (20%, v) and domestic sewage (80%, v) was fed into the MBR reactor, and over 95% COD and 50% NH_4_^+^-N were removed. One kind of amino acid similar to tryptophan was the prime reason that caused PTW resistance to be degraded. Analysis of the microorganism community in the MBR suggested that *norank_f__Saprospiraceae* was the key microorganism in degrading of PTW.

## 1. Introduction

The common methods used to treat pollutants included biological, physical, and chemical methods [1,2,3]. The biological method possesses the advantage of low cost [4]. However, it could not be applied directly to treat pharmaceutical tail water (PTW) because PTW is characterized by poor biochemistry and high toxicity [5]. Therefore, it is necessary for PTW to carry out pre-treatment prior to biological treatment.

Physical methods include coagulation, flotation, adsorption and membrane separation technology, etc. [6,7,8]. Among them, coagulation was widely used in the treatment of industrial wastewater and domestic sewage. Suspension solid and colloids could be mainly removed by coagulation. Polyaluminium ferric chloride (PAFC), polyaluminium chloride (PAC), and polyferric sulfate (PFS) were the most commonly used coagulants [9]. Chemical treatment of PTW has mainly used oxidants to open and break the bonds of organics with complex structures [10]. Fenton oxidation, one kind of common chemical process, generates hydroxyl radicals with strong oxidizing capacity in an acid solution [11]. Therefore, refractory organics can be transformed into some organic matters with low molecular weight and less toxicity. The advantages of Fenton oxidation were its ease of operation and rapid reaction rate [12]. Meanwhile, the ferric hydroxide complex generated at the end of the reaction would act as coagulant and enhance the removal efficiency of pollutants [13]. In recent years, the Fenton reaction has been widely applied to the treatment of refractory organic matters, and excellent performance has been observed [14]. Although the organic matters in PTW decreased after Fenton reaction, NH_4_^+^-N still could not be removed efficiently [15]. Previous studies showed that pre-treated PTW could be well treated by biological methods [16]. Notably, the membrane bioreactor (MBR) possesses excellent performance of sludge–water separation and produces less excess activated sludge in comparison with traditional biological methods [17].

Generally, the indicators for PTW treatment only include conventional chemical parameters such as COD and NH_4_^+^-N. However, due to the complexity of PTW, it is not possible to identify and analyze the tailwater components in depth. Therefore, statistical analysis can be used to analyze the parameters in PTW and identify the pollution components. Dissolved organic matter (DOM), as a sensitive indicator of water quality, can be used to reveal the chemical composition of PTW at various stages of physical, chemical, and biological treatment by excitation–emission-matrix spectra (EEM) and parallel factor analysis (PARAFAC), providing an in-depth understanding of the characteristics of PTW [18].

In the present study, the combined process of coagulation, Fenton reaction, and MBR was utilized to treat PTW. Firstly, the optimal coagulant, pH, and stirring program were determined through batch experiments and central composite design (CCD). Secondly, after the process of coagulation, the effect of several parameters, such as H_2_O_2_ dosage, FeSO_4_ dosage, pH, and reaction time on the removal of PTW by Fenton reaction was explored. Thirdly, the mixture of domestic sewage and pre-treated PTW was fed into the MBR reactor. The removal of COD and NH_4_^+^-N by MBR was investigated. Additionally, the details of the PTW removal process were analyzed by EEM, PARAFAC, and molecular weight distribution change. The variation in microorganism community in MBR was also explored. It was hoped that the present study could provide found data for the design of treatment of PTW.

## 2. Results

### 2.1. Characterization of PTW

The PTW was collected from an aerobic tank of a pharmaceutical factory in Liaoning Province and was stored at 4 °C before use. The tail water contains a high concentration of COD (209.8 mg/L), TOC (69.07 mg/L), NH_4_^+^-H (348 mg/L), and TN (1225 mg/L) with a low value of BOD/COD (0.06), indicating excess organic matters that were hard to be degraded.

**Figure 1:**
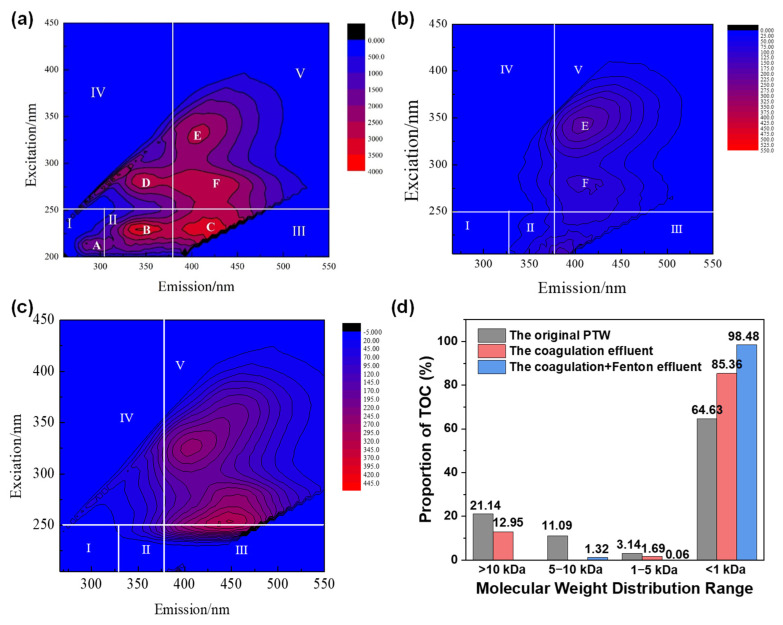
EEM spectra of (**a**) the original PTW, (**b**) coagulation effluent, (**c**) Fenton effluent, and (**d**) the molecular weight distributions of the original PTW, the coagulation effluent and co-process by coagulation and Fenton reaction effluent.

EEM of PTW was displayed in Figure 1a and can be divided into five regions (I, II, III, IV, and V). It was obvious that there were six fluorescence peaks (A, B, C, D, E, F), all of which could be found in river, sewage, landfill leachate, and coking wastewater. Peak A, located at Ex = 215 nm and Em = 300 nm, belonged to region I. The location of peak B was Ex = 235 nm and Em = 340 nm, corresponding to region II. Both peak A and peak B indicated protein and were related to tryptophan in dissolved organic matter (DOM), which often originates from human activities or microorganisms [19]. They were produced by microorganism metabolism [20]. Hence, peak A and peak B indicated to tyrosine and tryptophan, respectively. Peak C corresponded to region III and its location was Ex = 235 nm and Em = 420 nm [21]. It indicated fulvic acid and was related to the phenol and quinone group. Peak D corresponded to region IV and was located at Ex = 285 nm and Em = 345 nm. It indicated fulvic acid in an ultraviolet-like region, which possessed high levels of aromatization, high molecular weight, and was related to microorganism metabolism [22]. The excess of fulvic acid in the ultraviolet-like region caused low biological activity. Peak E corresponded to region V and was located at Ex = 330 nm and Em = 415 nm, indicating fulvic acid and humic acid in the visible light region [23,24]. Meanwhile, an obscure peak (Peak F) located at Ex = 275 nm and Em = 415 nm was observed, and it corresponded to region V. In general, the intensity of the fluorescence peak increased with the level of conjugation. Therefore, it can be confirmed that the DOM in the original PTW possessed excess conjugated double bond, and fulvic acid, tryptophan, as well as humic acid formed the predominant composition.

### 2.2. Research on Coagulation for PTW Treatment

#### 2.2.1. Effect of Coagulation on Treatment

**Figure 2:**
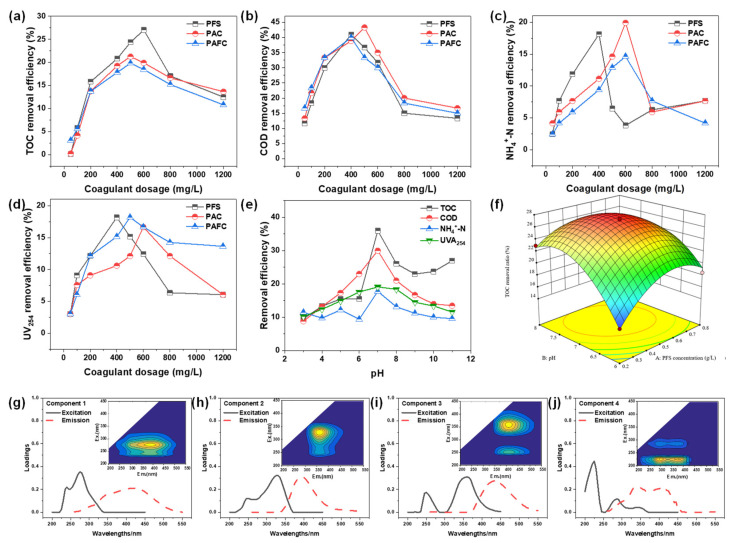
Effects of different coagulants on the removal efficiencies of (**a**) TOC, (**b**) COD, (**c**) NH_4_^+^-N, and (**d**) UV_254_ (pH = 7, dosages of coagulants were 0.05, 0.1, 0.2, 0.4, 0.6, 0.8, 1.0, and 1.2 g/L, 250 rpm for 2 min and 60 rpm for 10 min); (**e**) effects of initial pH on the removal efficiency of TOC, COD, NH_4_^+^-N, and UV_254_ of PTW treated by coagulation (pH = 3.0, 4.0, 5.0, 6.0, 7.0, 8.0, 9.0, 10.0, 11.0, 12.0, PFS = 400 mg/L, 250 rpm for 2 min and 60 rpm for 10 min); (**f**) 3D response surface graph for the removal of TOC with coagulation treatment; (**g**–**j**) EEM spectra of the PARAFAC components from coagulation effluent.

PFS, PAC, and PFAC were used as coagulants to treat the original PTW and the results were depicted in Figure 2a–d. Overall, the removal efficiencies of TOC, COD, NH_4_^+^-N, and UV_254_ initially increased and then decreased with the coagulants ranging from 0.05 g/L to 1.2 g/L. Consider the effect of PFS concentration on TOC removal efficiency as an example. In a neutral solution, PFS existed primarily in an anionic form (e.g., Fe (OH)_4_^−^ or Fe (OH)_3_ sol) and removed contaminants by sweeping net catch effect [25]. There was more anion of PFS at high PFS concentration (0.05–0.4 g/L). The generation of rolling flocculation took place easily, gaining better removal efficiency. Therefore, the optimal removal efficiency (22.7%) of TOC was achieved at 0.4 g/L PFS. However, the removal efficiency decreased when PFS concentration was above 0.4 g/L (0.4–1.2 g/L). It could be interpreted that excess anion generated by PFS at high PFS concentration would repel negatively charged colloids. Therefore, the removal efficiency of TOC declined. Additionally, the reason that the removal efficiency of TOC, COD, NH_4_^+^-N, and UV_254_ changed with PFS concentration may also be suitable for PAC and PAFC.

In Figure 2a, the optimal removal efficiency of TOC by PFS was superior to that by PAC and PFAC, and the corresponding values were 27.0%, 21.26%, and 19.83%, respectively. In Figure 2b, there was no obvious difference among the optimal removal efficiencies by the three coagulants. In Figure 2c, the optimal removal efficiencies followed the order PAC > PFS > PFAC. However, the optimum concentration of PAC and PFAC was 600 mg/L, which was much higher than that of PFS (400 mg/L). From Figure 2d, it can be seen that the optimal removal efficiency of PFS, PAC, and PFAC toward UV_254_ was almost the same (17.44% ± 0.77%). In summary, PFS was superior to PAC and PFAC for its low dosage and high removal efficiency. Hence, PFS was used as coagulant in further study.

#### 2.2.2. Effect of pH on PTW After PFS Treatment

The effects of initial pH on the removal efficiencies of TOC, COD, NH_4_^+^-N, and UV_254_ of PTW treated by coagulation were displayed in Figure 2e. It was observed that the removal enhanced and then decreased as the pH increased from 3.0 to 11.0. For example, the removal of TOC at pH 3.0, 7.0, and 11.0 were 9.6%, 44.9%, and 26.9%, respectively. For TOC, COD, NH_4_^+^-N, and UV_254_, all the optimal removal efficiencies were achieved around pH 7.0, and the corresponding values were 44.9%, 30.0%, 16.0%, and 17.3%, respectively. This could be explained by the following reasons. First, PFS exists mainly in a cationic form at low solution pH and removes pollutants by electric neutralization and adsorption bridging [26]. Meanwhile, colloids in an aqueous solution with low solution pH were surrounded by excess H^+^, impeding the interaction between Fe^3+^ and colloids [27]. Therefore, low removal efficiency was observed. However, at relatively higher pH, stable coagulation between PFS, H^+^, and colloids would be rapidly formed [28]. They would be isolated from the aqueous solution and would become conducive to the occurrence of adsorption bridging, leading to better removal efficiency. PFS would exist mainly in anion form and would remove pollutants by sweeping net capture effect when aqueous solution contained higher concentration of OH^−^, while excess anion hindered the formation of a stable system [29]. Accordingly, the plateau of removal efficiency was observed at pH 7.0.

#### 2.2.3. Optimization and Analysis After Coagulation Treatment

Based on the CCD method, the responses of TOC removal to initial pH and PFS concentration were studied, and results were given in Tables S1 and S2. Related model was established: TOC removal efficiency = −220.318 + 79.1458A + 62.501B − 5.27725AB − 39.9224A^2^ − 4.10671B^2^. To clarify the significance level of each factor’s influence on TOC removal, analysis of variance (ANOVA) was employed for evaluation. A *p*-value < 0.05 confirmed the validity of the response model, with close agreement between predicted and experimental values, demonstrating robust reliability. As shown in Table S3, the value of F in the model was 58.56, and *p* was under 0.0001. It indicated that the adaptability of the model was significant. The coefficient of determination (R^2^) and adjusted coefficient of determination (R^2^_adj_) were 0.9336 and 0.9803, respectively. The difference between R^2^ and R^2^_adj_ was 0.0467, which was less than 0.2, indicating that the model possesses high credibility and precision.

Figure 2f displayed the results of response surface analysis. The response surface has a steeper slope on the B-pH side, with contour lines intersecting the B-pH more densely than the A-PFS, indicating a greater inclination along the B-pH. According to the distribution plot of desirability function values in Figure S2, The TOC removal rate was taken as the optimization objective, and the maximum TOC removal rate was taken as the optimization objective. The desirability function with a value ranging from 0 to 1 was used to evaluate the quality of the optimization results. It can be seen that the theoretically optimal TOC removal efficiency (27.4%) was achieved at 0.53 g/L PFS and pH 7.25. The value was close to the experiment value (25.6%) with a deviation of 6.57%. It demonstrated that the prediction of the response surface model about TOC removal was accurate.

#### 2.2.4. Effect of Stirring Procedure on Coagulation

The effects of various coagulation programs on removal efficiency of TOC were carried out by adjusting stirring time and speed. The results are shown in Table S4.

From programs 1, 2, and 3, it can be seen that the removal efficiency of TOC increased slowly with the operation time (1–3 min) of rapid stirring. This was because that adsorption bridge and sweeping net capture effect would enhance with the extension of time when the mixer stirred at 250 rpm.

Comparing programs 1, 4, and 5, it was obvious that the removal efficiency of TOC remained almost constant (22.13% ± 0.31%) when the time for slow stirring ranged from 5 min to 15 min. A previous study also suggested that excessive time of slow stirring would cause the flocculant to dissolve, while the flocculant was not completely precipitated within short time [30].

From programs 1, 6, and 7, it could be concluded that the holding time was an important parameter for TOC removal efficiency. The removal efficiencies were 21.1% and 22.4% when the holding time were 0.5 h and 1 h, respectively. While it was only 8.9% when the holding time was 2.0 h. It indicated that the precipitation process took place in the first 30 min and the lower removal efficiency was caused by the dissolution of flocculation when it was stood for a long time.

In programs 1, 8, and 9, the removal efficiency of TOC was highly related to the speed of rapid stirring, while other parameters were kept constant. The corresponding removal efficiencies were 14.46%, 24.46%, and 16.74% when the stirring speeds were 150 rpm, 250 rpm, and 350 rpm, respectively. This was because the rapid stirring destroyed the flocculation, while the coagulant did not have sufficient contact with pollutants at low stirring speeds.

The slight difference in removal efficiency in programs 1, 9, and 10 indicated that the operation time (30–90 min) of slow stirring had an insignificant influence.

#### 2.2.5. Coagulant on Remove of DOM

(1) Analysis of EEM

EEM was used to confirm the removal of DOM by coagulation and the results are given in Figure 1b. Compared with the peak of the original PTW, both the peak type and intensity decreased in the EEM of the effluent. The disappearance of peaks A, B, C, and D may be caused by two reasons. First, some organic matters, such as macromolecular organic matters, aromatic compound, and hydrophobic compound with saturated double bond were removed [31]. Second, some fluorophores were overlapped [32]; for example, peak E located at Ex = 375 nm and Em = 415 nm, and peak F located at Ex = 280 nm and Em = 415 nm. Both peak E and peak F were in region V, which was the sign of fulvic acid and humic acid in the visible light region. It indicated that fulvic acid and humic acid in PTW resist being removed.

(2) PARAFAC analysis of coagulation effluent

As described above, the fluorescent peak could not be determined accurately by traditional methods if fluorophore was overlapped or moved. Thus, PARAFAC was used to confirm the component of coagulation effluent of PTW and the results were obtained when four kinds of components were distinguished (Figure 2g–j). The location of Compound 1 was Ex = 275 nm and Em = 425 nm, indicating humic acid and fulvic acid in the visible light region. It was related to some matters that resisted being degraded with macromolecular and a high degree of aromatization, such as polycyclic aromatic hydrocarbons [33]. However, up to data, the composition of Component 1 was still unclear because the corresponding peak was absent in the EEM of the original PTW as well as the coagulation effluent. Accordingly, there were two reasons that may explain the phenomenon. First, the residual coagulant interfered with the expression of fluorescent matters [34]. Second, new intermediates were formed in the coagulation process [35]. The peak of Component 2 was located at Ex = 325 nm and Em = 400 nm, which belonged to region V. It indicated that Component 2 was UVA humic humus and possessed small molecules. It was also concluded that the matters removed by the coagulation process were mainly macromolecular organics. Component 3, a common high molecular weight humus belonging to UVC humic humus, was located in region V and the location was Ex = 355/250 nm and Em = 435 nm. The position of the fluorescence peak of Component 4 (Ex = 230 nm and Em = 335 nm) was placed in region II, meaning that Component 4 was a tryptophan-like substance [36]. The decreased signal intensity of the fluorescence peak indicated that some tryptophan-like substances have been removed.

(3) Distribution of molecular weight

The molecular weight distributions of the original PTW and the coagulation effluent were displayed in Figure 1d. As can be seen, the percentage of organics with molecular weight larger than 10 kDa in the original PTW (21.14%) was higher than that in the coagulation effluent (12.95%). This situation was also suitable for organics with molecular weight 5–10 kDa and 1–5 kDa. However, it was found that the percentage of organics with a molecular weight of less than 1 kDa in the original PTW (64.63%) was lower than that in the coagulation effluent (85.36%). It indicated that the organics removed in the coagulation process were mainly macromolecular organic matters and that some macromolecular organic matters transferred into small molecular matters.

### 2.3. The Degradation of Effluent of Coagulation by Fenton Reaction

#### 2.3.1. The Effects of Various Parameters on TOC Removal Efficiency of PTW

**Figure 3:**
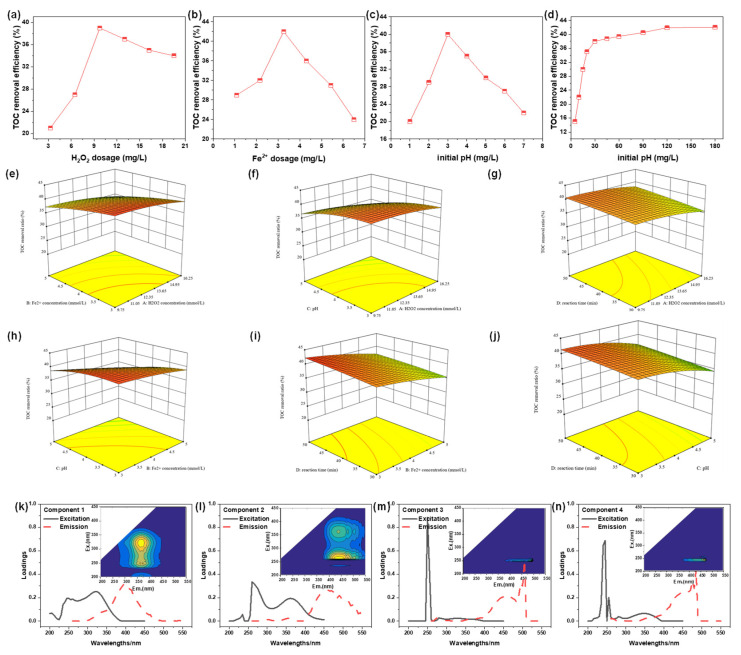
The influence of H_2_O_2_ dosage (**a**), FeSO_4_ concentration (**b**), initial solution pH (**c**), and reaction time (**d**) on TOC removal efficiency of pharmaceutical park tail by Fenton reaction. (FeSO_4_ = 3.25 mmol/L, H_2_O_2_ = 9.75 mmol/L, pH = 3 and T = 120 min unless special note); (**e**–**j**) 3D response surface graph for the removal of TOC after Fenton treatment; (**k**–**n**) EEM spectra of the PARAFAC component from Fenton effluent.

The effects of some key parameters, such as FeSO_4_ and H_2_O_2_ concentration, initial solution pH, and reaction time on the TOC removal efficiency of PTW were investigated by batch experiments and the results were shown in Figure 3a–d. From Figure 3a, it can be seen that the removal efficiency increased with H_2_O_2_ concentration until the maximum value (36.9%) was obtained at 9.75 mmol/L H_2_O_2_, then it slowly declined with increasing H_2_O_2_ concentration, which was consistent with the previous study [37]. This was due to the fact that more ·OH, a power oxidant with redox potential of −2.0 eV, was generated at higher H_2_O_2_ concentration, while excess H_2_O_2_ consumed ·OH and ·HO_2_ was formed. Compared to ·OH, ·HO_2_ exhibited weaker oxidation capacity [38]. Accordingly, the removal efficiency decreased with excess H_2_O_2_. In Figure 3b, the influence of FeSO_4_ concentration on TOC removal efficiency was similar to that of H_2_O_2_ concentration, and the optimum FeSO_4_ concentration was 3.25 mmol/L with the corresponding removal of 41.8%. The increased FeSO_4_ dosage would promote the generation of ·OH within moderate FeSO_4_ concentration. However, excess Fe^2+^ would terminate ·OH with pollutants, although increased Fe (OH)_2_ also improved the coagulation effect [39].

Figure 3c indicated that the optimum pH for the degradation of PTW by Fenton reaction was 3.0, and 38.9% of TOC was removed. In fact, Fe^2+^ existed mainly in the form of [Fe(II)(H_2_O)_6_]^2+^, which has weaker catalytic capacity for H_2_O_2_ compared to [Fe(II)(OH)(H_2_O)_5_]^+^ [40]. Therefore, fewer ·OH was generated in the strong acid solution. However, Fe^2+^ easily precipitates at high OH^−^ concentration. The weaker catalytic capacity of the precipitates resulted in decreased TOC removal efficiency.

In Figure 3d, the TOC removal efficiency increased rapidly with the reaction time in the first 30 min and then increased slowly with the extension of time (30–120 min). The removal efficiencies at 30 and 120 min were 38.3% and 41.2%, respectively. Their faint difference indicated that 30 min was the optimal reaction time.

#### 2.3.2. Optimization and Analysis After Fenton Treatment

Based on the CCD method, the responses of the TOC removal to Fe^2+^ and H_2_O_2_ concentration, solution pH, and reaction time were carried out and the results are given in Tables S5 and S6. The regression analysis can be expressed as follows: TOC removal efficiency = −1.15703 + 1.67791A + 6.74066B + 12.2586C + 0.126197D − 0.0324519AB − 0.111202AC + 0.00767788AD − 1.06297BC − 0.0134531BD + 0.0141406CD − 0.0711638A^2^ − 0.490077B^2^ − 1.20917C^2^ − 0.00168799D^2^. ANOVA analysis of TOC removal in Table S3 showed that the value of F in model was 33.65 and *p* was under 0.0001. It indicated that the model was highly adaptable to the results. Meanwhile, the small difference (0.0811) between R^2^ (0.8592) and R^2^_adj_ (0.9403) indicated that the model possessed high credibility and accuracy.

Response surface analyses and contour plots were given in Figure 3e–j. It can be seen that the interaction between B-Fe^2+^ dosage and C-pH exhibited the steepest response surface, with the most complex variation pattern, indicating the most significant interactive influence between B and C. Additionally, the response surface shows varying degrees of change across its entire range, suggesting that optimizing Fe^2+^ concentration and pH can most effectively enhance the removal efficiency. According to the distribution plot of desirability function values in Figure S3, optimal TOC removal efficiency (42.79%) was achieved at H_2_O_2_ = 11.6 mmol/L, FeSO_4_ = 3.0 mmol/L, pH = 3.3 and T = 50 min. The theoretical value was close to the experiment result (43.1%) obtained under the same condition.

#### 2.3.3. Analysis of DOM After Fenton

(1) Analysis of EEM spectra

The EEM of the Fenton reaction effluent was displayed in Figure 1c, and two fluorescence peaks were observed in region V. Peak A, located at Ex = 325 nm and Em = 410 nm, was usually detected in agricultural wastewater [41]. In the present study, it could be speculated that peak A implied some refractory heterocyclic organics [42]. The position of peak B was Ex = 250 nm and Em = 450 nm. It indicated some macromolecular amino acid humus, the content of which was higher in wetlands and forests. Compared with Figure 1a,b, both the intensity and the type of the fluorescence peak indicating refractory and high degree of humification organics declined. It could be concluded that Fenton reaction degraded most of the organic compounds contained in PTW. Meanwhile, some macromolecular organics were transformed into small molecular ones and the refractory ones became easier to degrade [43].

(2) PARAFAC analysis of Fenton effluent

To further characterize the component of DOM of Fenton reaction effluent, PARAFAC was used to analyze EEM and the results were shown in Figure 3k–n. Component 1, which was located at Ex = 320 nm and Em = 400 nm, was usually found in agricultural wastewater. Component 2 exhibited two obvious peaks, named Component 2-1 (C2-1) and Component 2-2 (C2-2). For C2-1, its location was Ex = 355 nm and Em = 460 nm, indicating macromolecular humus. While for C2-2, its location was Ex = 355 nm and Em = 460 nm, which implied aromatic amino acid humic substances. Component 3 was located at Ex = 250 nm and Em = 500 nm, and it was a common macromolecular aromatic amino acid humic compound and shared a similar fluorescence feature with fulvic acid. The location of Component 4 was close to that of Component 3, and Component 4 was also an aromatic amino acid humic compound. However, the fluorescence intensity of Component 4 was lower than that of Component 3, indicating that Component 3 mainly caused a high concentration of TOC of the Fenton reaction effluent.

(3) Analysis of Molecular Weight Distribution

As displayed in Figure 1d, the percentage of organics with molecular weight >10 kDa after the Fenton reaction was 0.14%, and that in the coagulation effluent was 12.95%. Meanwhile, the percentage of organics with molecular weight < 1 kDa increased from 85.36% to 98.48% after the Fenton reaction. It implied that some macromolecular organics were degraded by Fenton degradation. The increased percentage (0–1.32%) of organics with molecular weight 5–10 kDa may be attributed to intermediates or the complex between ferrous ion and some organics.

### 2.4. Treatment of PTW After MBR

#### 2.4.1. Removal of COD and NH_4_^+^-N by MBR

**Figure 4:**
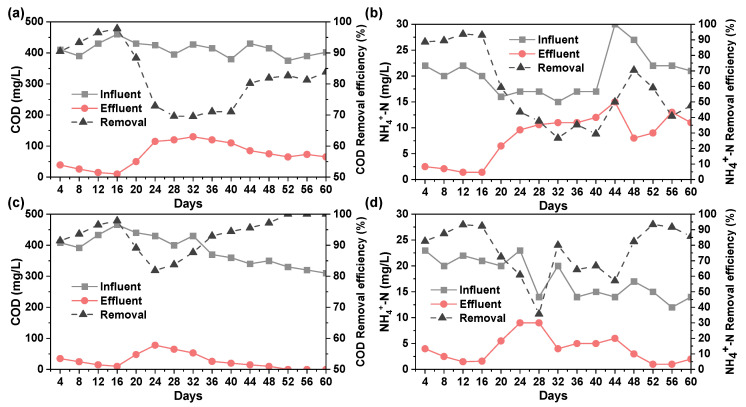
Changes in COD and NH_4_^+^-N removal from (**a**,**b**) MBR-A and (**c**,**d**) MBR-B.

The removal of COD and NH_4_^+^-N in the mixture of domestic sewage and the original PTW by MBR-A was exhibited in Figure 4a,b. It can be seen that the removal efficiencies of COD and NH_4_^+^-N increased with the reaction time within the first 16 days and then declined on the 16th day due to the percentage of PTW in the influent increasing to 50%. The increased concentration of PTW increased the toxicity to microorganism and broke the balance of the reactor, causing lower reactivity activity. The observed improved COD removal efficiency on the 20th day may be caused by the natural degradation of simulated domestic sewage. The COD concentration on the influent decreased and that in the effluent decreased accordingly. Due to the change in water quality, the microorganisms in the MBR reactor could not adapt to the new environment within a short period of time [44]. Accordingly, the removal efficiency of COD and NH_4_^+^-N fluctuated acutely until the 40th day. Ultimately, the effluent contained 65 mg/L COD and 11.4 mg/L NH_4_^+^-N.

The degradation of the mixture of domestic sewage and PTW effluent after Fenton reaction was carried out in MBR-B. As was displayed in Figure 4c,d, the removal efficiency of COD and NH_4_^+^-N declined when the percentage of PTW increased to 50%, which was similar to that in MBR-A. However, the microorganisms in MBR-B adopted to the new environment in a shorter time compared to that in MBR-A. For example, the COD removal efficiency in MBR-A tends to stabilize on the 24th day, while it took only 8 days in MBR-B. This can be explained by the fact that the concentration of refractory organics in the influent of MBR-B was lower than that in MBR-A after coagulation and Fenton reaction. Therefore, MBR-B suffered less adverse effects. Ultimately, the NH_4_^+^-N in the effluent of MBR-B was 7.8 mg/L, and the COD was almost completely degraded.

#### 2.4.2. Analysis of DOM After Treatment

(1) PRAFAC analysis of MBR-A influent and effluent

**Figure 5:**
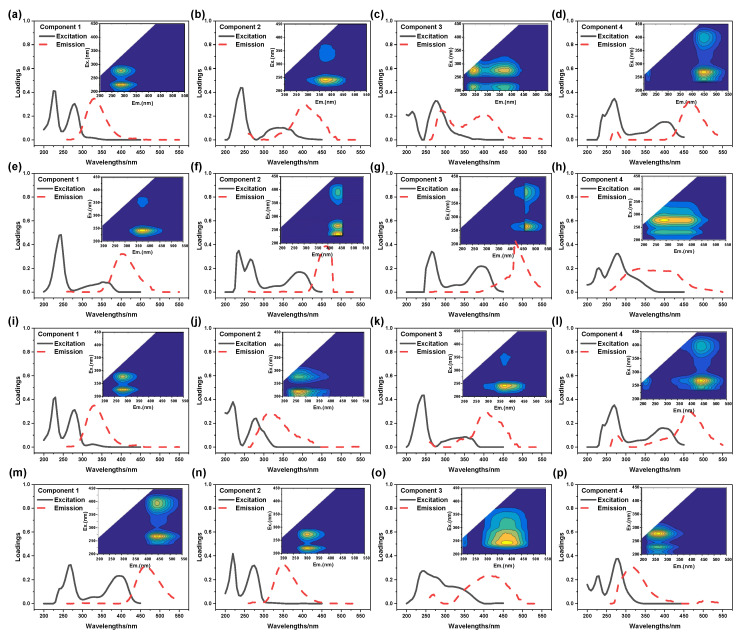
EEM spectra of the PARAFAC component from (**a**–**d**) MBR-A influent, (**e**–**h**) MBR-A effluent, (**i**–**l**) MBR-B influent; (**m**–**p**) MBR-B effluent.

The DOM component of influent in MBR-A was analyzed by PARAFAC and the results were given in Figure 5a–d. It can be seen that Component 1 exhibited two fluorescence peaks. One peak was located at Ex = 225 nm and Em = 338 nm, while the other was located at Ex = 275 nm and Em = 338 nm. Both of them indicated protein in the UV zone and were related to the metabolism of microorganisms [45]. Component 2, a common macromolecule humus, was located at Ex = 240 nm and Em = 405 nm. The location of Component 3 was Ex = 275 nm and Em = 400 nm. Component 4 was located at Ex = 400 nm and Em = 470 nm and was a component of aromatic protein humus in the UV region.

Figure 5e–h displayed the analysis results of the composition of the effluent in MBR-A. As can be seen, Component 1 was located at Ex = 240 nm and Em = 405 nm. Its location and peak intensity were almost the same as that of Component 2. It indicated that macromolecule humus was the key to PTW degradation. The locations of Component 2, Component 3, and Component 4 were similar to the corresponding component in the influent. The slight shift in the location of the fluorescence peak implied that the polarity and structure of the organics in the influent changed when PTW was treated by MBR-A [46].

(2) PARAFAC analysis of MBR-B influent and effluent

The composition of the influent in MBR-B was depicted in Figure 5i–l. Component 1 was located at Ex = 275/225 nm and Em = 330 nm and belonged to amino acid substance. Component 2 was located at Ex = 275 nm and Em = 310 nm, and it was part of lysine. Component 3 was located at Ex = 240 nm and Em = 400 nm. It was humic humus and was often detected in wastewater. Component 4, a macromolecule of aromatic amino acid humus, was located at Ex = 260/400 nm and Em = 470 nm.

The component of the effluent in MBR-B was exhibited in Figure 5m–p. As can be seen, the location of Component 1 was Ex = 260/400 nm and Em = 460 nm, which was similar to Component 4 of the influent in MBR-B. Meanwhile, the intensity of Component 1 was also close to that of Component 4, which indicated that macromolecule aromatic amino acid humus was resistant to being degraded in MBR-B. Component 2 was located at Ex = 225/275 nm and Em = 350 nm, and it was one of the amino acids that was dissociated or combined with a protein. Compared with the reported literature, C1 content is dependent on bound extracellular polymeric substance (bEPS) amounts in sludge [47]. Component 3, a low molecular weight compound, was located at Ex = 290 nm and Em = 410 nm. It indicated that most macromolecule organics were transformed into small molecular organics by MBR-B. Component 4, an amino acid which was similar to tryptophan, was located at Ex = 225/275 nm and Em = 330 nm [48]. It was dissociated or bound to proteins and was found in both the influent and effluent of MBR-A and MBR-B. Therefore, it could be speculated that Component 4 was one of the refractory organics contained in PTW.

#### 2.4.3. Comparison of the Microorganism Diversity Between MBR A and B

(1) Microorganism diversity index analysis

The results of microorganism diversity index analysis are listed in Table S8. Sample C was the microorganism community at the initial stage. Sample AZ and MBR-AZ denoted the microorganism community in the middle and final stages of MBR-A, respectively. Sample BZ and MBR-BZ represented the microorganism community in the middle and final stages of MBR-B, respectively. It can be seen that all the coverage indexes were larger than 0.99, which implies that the results were highly reliable. The Shannon index among the five samples followed the order of AZ > C >MBR-BZ > BZ> MBR-AZ. Accordingly, the microorganism species of AZ was the highest among the five samples. The ace and chao index represented the total number of microorganisms [49]. Therefore, it can be speculated that the number of microorganisms among the five samples followed the order of C > BZ > MBR-BZ > AZ > MBR-AZ.

(2) Analysis of community structure and succession

**Figure 6:**
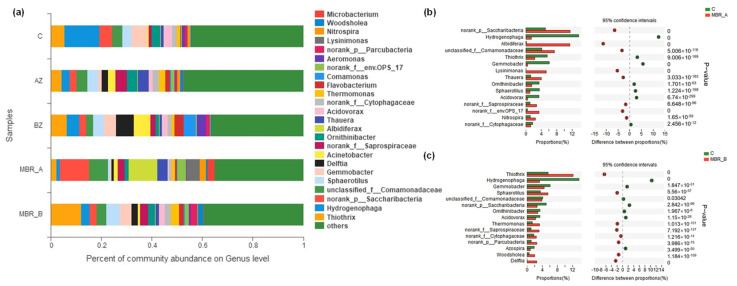
(**a**) Community histograms based on species level; species differences before and after operation in (**b**) MBR-A reactor and (**c**) MBR-B reactor.

In order to study the community structure and succession when activated, sludge was inoculated into the MBR reactor, the distribution of microorganism (>2%) based on genus lever was studied. As can be seen in Figure 6a, the percentage of *Nitrospira* in MBR-B was higher than that in MBR-A. This was one of the reasons why the removal efficiency of NH_4_^+^-N in MBR-B was superior to that in MBR-A. Meanwhile, the number of *hydrogenophaga* seriously decreased with the reaction time. It was because *hydrogenophaga* was one of the Gram-negative bacteria, which utilized amino acid and protein as nutrients, but used less glucose. However, amino acid and protein were transformed into small molecular substances with the extension of reaction time, leaving fewer available nutrients. Compared with other samples, the percentage of *Noran_k__Saprospiraceae* and *Albidiferax* obviously increased and became the dominant species. Combined with a literature analysis, it can be speculated that *Noran_k__Saprospiraceae* and *Albidiferax* were the key microorganisms in the degradation process of PTW [50].

(3) Comparative analysis of community

The difference in microorganism species between C and MBR-A is displayed in Figure 6b. It was observed that the number of *norank_p__Saccharibacteria*, *Albidiferax*, *unclassified_f__Comamonadaceae*, *Lysinimonas*, *Thauera*, *norank_f__Saprospiraceae*, *norank_f__env.OPS_17* and *Nitrospira* increased with reaction time, indicating that those bacteria adopted to the high salt environment of PTW and played an important role in the degradation process [51]. However, the number of *Hydrogenophaga*, *Thiothrix*, *Ornithinibacter*, *Sphaerotilus*, *Acidovorax* and *norank_f__Cytophagaceae* declined with the reaction time. It implied that these microorganisms could take use of PTW as energy.

The amount of *Thiothrix*, *Sphaero-tilus*, *Thermomonas*, *norank_f__Saprospiraceae*, *norank_f__Cytophagace-ae*, *norank_p__Parcubacteria*, *Woodsholea,* and *Delftia* in MBR-B was higher than that in C (Figure 6c). It means that these bacteria promoted the degradation of PTW. The declination of *norank_p__Sac–charibacteria*, *Albidiferax*, *unclassified_f__Comamonadaceae*, *Lysinimonas*, *Thauera,* and *norank_f__env.OPS_17* denoted that these microorganisms could not be degraded well in PTW.

Comparing the change in abundance between MBR-A and MBR-B, it was obvious that the abundance of *norank_f__Saprospiraceae* increased in both MBR-A and MBR-B. Therefore, it could be speculated that *norank_f__Saprospiraceae* was the key microorganism in the degradation of PTW. The amount of *norank_p__Sac–charibacteria*, *Albidiferax*, *unclassified_f__Comamonadaceae*, *Lysinimonas*, *Thauera,* and *norank_f__env.OPS_17* increased in MBR-A, but declined in MBR-B. Meanwhile, the removal efficiency of NH_4_^+^-N in MBR-B was better than that in MBR-A. Therefore, it can be speculated that these bacteria possess a weak capacity to utilize NH_4_^+^-N.

## 3. Materials and Methods

### 3.1. Materials

In this study, all the reagents included, polyferric sulfate (PFS), polyaluminium chloride (PAC), polyaluminium ferric chloride (PAFC), sodium hydroxide (NaOH), hydrogen chloride (HCl), sulfuric acid (H_2_SO_4_), hydrogen peroxide(H_2_O_2_, 30%, w), ferric sulfate heptahydrate (FeSO_4_·7H_2_O), sodium oxalate (HCO_3_COONa), urea (CO(NH_2_)_2_), ammonium chloride (NH_4_Cl), calcium chloride (CaCl_2_), magnesium sulfate (MgSO_4_), dipotassium hydrogen phosphate (KH_2_PO_4_), sodium bicarbonate (NaHCO_3_), and glucose (C_6_H_14_O_7_), were purchased from Sinopharm Chemical Reagent Co., Ltd. (Shanghai, China). with analytical purity.

### 3.2. Methods

#### 3.2.1. Treatment of PTW by Coagulation

PAC, PFS, and PAFC were used as coagulants to treat PTW at 20 ± 2 °C. The experiments were conducted on a mixer (ZR4-6, Zhongrun Water industry technology development Co., Ltd., Shenzhen, China). Typically, 1 L of PTW was added to a cylindrical plexiglass container (1.4 L), and the pH of the solution was adjusted by 1 mol/L HCl and NaOH. The mixer was started when a certain amount of coagulant was accurately weighed by an electronic balance (ML54, Mettler, Columbus, OH, USA) and added to the container. At a predetermined time, the mixer was stopped, and the supernatant was used to measure the concentration of TOC, COD, NH_4_^+^-N, and UV_254_ after 1 h. Three parallel experiments were performed for all the above experimental procedures.

#### 3.2.2. Treatment of Effluent of Coagulation Process by Fenton Reaction

The degradation of PTW by Fenton reagents was conducted in a 0.8 L cylindrical plexiglass container with a height of 13.5 cm and a diameter of 3.3 cm at 20 ± 2 °C. The experimental procedure was as follows. Firstly, 0.5 L of effluent of coagulation reaction was added to the container and the pH of the solution was adjusted by 1 mol/L HCl and NaOH. Then the mixer was started, followed by the addition of a certain amount of FeSO_4_ and H_2_O_2._ Samples were taken from the container at a fixed time and their solution pH was adjusted to 10 by 1 mmol/L NaOH immediately to terminate the reaction. The supernatant was obtained when the sample was left in the water bath for 30 min. Before analysis, supernatant was filtered through 0.45 μm cellulose acetate membrane. All experimental procedures were performed three times in parallel.

#### 3.2.3. Treatment of Mixture of PTW and Domestic Sewage by MBR

The experiment was conducted in a cylindrical plexiglass container with an available capacity of 5 L. The inner diameter and height of the container were 8 mm and 25 mm, respectively. The hollow fiber membrane used in this study was produced by Beijing Origin Water Technology Co., Ltd. (Beijing, China). The area of the membrane was 2 m^2^ and the sizes of A, B and C were 0.1 μm, 0.9 mm and 1.5 mm, respectively. The reactor was operated sequentially, and the flow rate (600 mL/h) was controlled by a peristaltic pump, and the hydraulic residence time was 10 h.

The activated sludge was obtained from the secondary clarifier of a wastewater treatment plant in Beijing. The concentration and settling velocity (SV) of sludge were 3400 mg/L and 30%, respectively. The supernatant was discarded when the activated sludge had stood for 2 h and the remaining precipitate had been aerated for 12 h, followed by the addition of 4 L of simulated domestic wastewater (400 mg/L C_6_H_14_O_7_, 200 mg/L HCO_3_COONa, 20 mg/L CO(NH_2_)_2_, 80 mg/L NH_4_Cl, 20 mg/L KH_2_PO_4_, 250 mg/L CaCl_2_, 300 mg/L MgSO_4_ and 100 mg/L NaHCO_3_) with constant aeration. From the next day, the influent flow rate was gradually increased to the designed value until SV and the concentration of sludge reached 30% and 3000 mg/L, respectively. Then, the sludge was inoculated into the MBR reactor. The mixture of PTW (10%, v) and domestic sewage (90%, v) was added to the reactor when the water quality of the effluent was kept constant. The percentage of PTW increased to 20% on the eighth day since the mixture was added into the MBR reactor. Especially, as shown in Figure S1, the PTW added into reactor A was the original one, while that added into reactor B was the effluent of the Fenton reaction. Samples were withdrawn every 2 days when the mixture was added.

The changes in microorganism population and community were determined using high throughout sequencing technology. Firstly, 500 mL sludge stood for 30 min and 50 mL precipitation was collected and placed in 50 mL sterile centrifuge tube, then centrifuged for 10 min at 3000 r/min by 6–10 °C. Then, 5 mL sludge was collected and used for DNA extraction. The extraction of DNA was performed according to the previous literature.

DOM in water was detected by EEM using a fluorescence spectrometer (F-7000, Hitachi, Tokyo, Japan). Quinine sulfate solution (4 μg/L dissolved in 0.05 mol/L sulfuric acid) was used to correct the fluorescence intensity before analysis. During sample pretreatment, the water sample should first be diluted. Milli-Q ultra-pure water was used as a blank control to eliminate the influence of Raman scattering, and the value of EEM was removed from the blank control value. Inner-filter effects and Rayleigh scatter effects were eliminated during data processing.

#### 3.2.4. Statistical Analysis

Modeling and optimization of TOC removal efficiency by coagulation and effluent were adopted by single factor analysis and response surface methodology. CCD was applied using the software Design-Expert (version 13.0). In this study, we set initial pH and PFS concentration in the responses of TOC removal by coagulation process, and Fe^2+^, H_2_O_2_ concentration, solution pH, and reaction time in the responses of TOC removal by effluent process. The relationship between the input variables and the output response can be expressed by the quadratic model:

(1) $$Y\left(X1\right)=a_{0}+\sum_{i=1}^{n}a_{i}X_{i}+\sum_{i=1}^{n}b_{i}X_{i}^{2}+\sum_{i=1}^{n}\sum_{j=1}^{n}c_{ij}X_{i}X_{j}$$

where *Y*(*X*) is the predicted percentage of TOC removal, *Xi* are the studied independent factors, *a*_0_ is the constant coefficient, *a_i_* are the linear coefficient, *b_i_* are the square coefficients, and *c_ij_* are the interaction coefficients.

PARAFAC was applied to analyze EEM data with DOM fluor based on MATLAB 2017a.

## 4. Conclusions

In this study, the original PTW was treated by the combined process of coagulation, Fenton reaction and MBR reactor. The main results were as follows:

(1) Compared to PAC and PAFC, PFS possessed the advantage of lower dosage and higher removal efficiencies to TOC, COD, NH_4_^+^-N, and UV_254_ of the original PTW_._ The optimum pH and PFS dosage were 7.25 and 0.53 g/L, respectively. Batch experiment indicated that the best performance of coagulation process was achieved when the mixer was stirred at 250 rpm for 3 min and 60 rpm for 10 min, followed by being left for 60 min. According to the difference in molecular weight distribution between the before and after coagulation process, the matter mainly removed by coagulation process was the organics with molecular weight above 10 kDa.
(2) For PTW treated by coagulation process, the best removal efficiency of TOC (43.1%) was obtained by Fenton reaction at 11.6 mmol/L H_2_O_2_, 3.0 mmol/L FeSO_4_, pH = 3.3 and T = 50 min. A kind of common macromolecule aromatic amino acid compounds located at Ex = 250 nm and Em = 500 nm was the main reason that caused the high TOC concentration of the effluent. The decreased percentage of organics with molecular weight above 10 kDa and the increased percentage of organics with molecular weight below 1 kDa meant that some macromolecule compounds were transformed into small molecular weight compounds by Fenton reaction.
(3) Compared with the original PTW, the one treated by coagulation process and Fenton reaction could achieve stable removal efficiency of COD and NH_4_^+^-N in a shorter time. More than 95% COD and 50–60% NH_4_^+^-N were removed when the mixture of pre-treated PTW and domestic sewage was used as an influent. According to EEM, a kind of amino acid which was similar to tryptophan was the main reason that caused PTW resistance to be degraded. Analysis of the microorganism community in the MBR suggested that *norank_f__Saprospiraceae* was the key microorganism in the degradation of PTW.

The study results will provide a more efficient and precise method for analyzing and managing water components in the deep treatment of PTW. Nevertheless, further studies should be conducted to correlate organic molecules with DOM to seek in-depth application of DOM analysis in treatment of PTW.

## Data Availability

Data are contained within the article and Supplementary Materials.

## Supplementary Materials

The following supporting information can be downloaded at: https://www.mdpi.com/article/10.3390/molecules30122520/s1, Figure S1: Schematic diagram of the experimental design. Figure S2: Distribution plot of desirability function values. Figure S3: Distribution plot of desirability function values. Table S1: Experimental factor levels. Table S2: Design matrix for the two independent variables and the corresponding results at different experimental levels. Table S3: ANOVA analysis of the TOC removal. Table S4: Effect of Different Procedures on Stirring. Table S5: Experimental factor levels. Table S6: Design matrix for the independent variables and the corresponding results at different levels. Table S7: ANOVA analysis of the TOC removal. Table S8: Microorganism diversity index analysis.
