# Supplementary material for: Fate of Dissolved Organic Matter and Cooperation Behavior of Coagulation: Fenton Combined with MBR Treatment for Pharmaceutical Tail Water
Source: Molecules. 2025 Jun 9;30(12):2520. doi: 10.3390/molecules30122520 (PMC12196186; doi:10.3390/molecules30122520)
Supplement: Supplementary file 1 [file molecules-30-02520-s001.zip › molecules-3621900-supplementary.pdf]

Supporting Information for

Fate of Dissolved Organic Matter and Cooperation behavior of Coagulation, Fenton Combined with MBR treatment for Pharmaceutical Tail Water

Jian Wang[1, 2], Chunxiao Zhao[1], Feng Qian[1, 3*], Jie Su[1, 2*], Hongjie Gao[1, 3]

[1]*State Key Laboratory of Environmental Criteria and Risk Assessment, Chinese Research Academy of Environmental Sciences, Beijing 100012, China*

[2]*Key Laboratory of Estuarine and Coastal Environment of the Ministry of Ecology and Environment, Chinese Research Academy of Environmental Sciences, Beijing 100012, China*

[3]*Institute of Water Eco-environment Research, Chinese Research Academy of Environmental Sciences, Beijing 100012, China*

[*] Corresponding authors.

E-mail address: qianfeng@creas.org.cn (Feng Qian); sujie16@126.com (Jie Su)

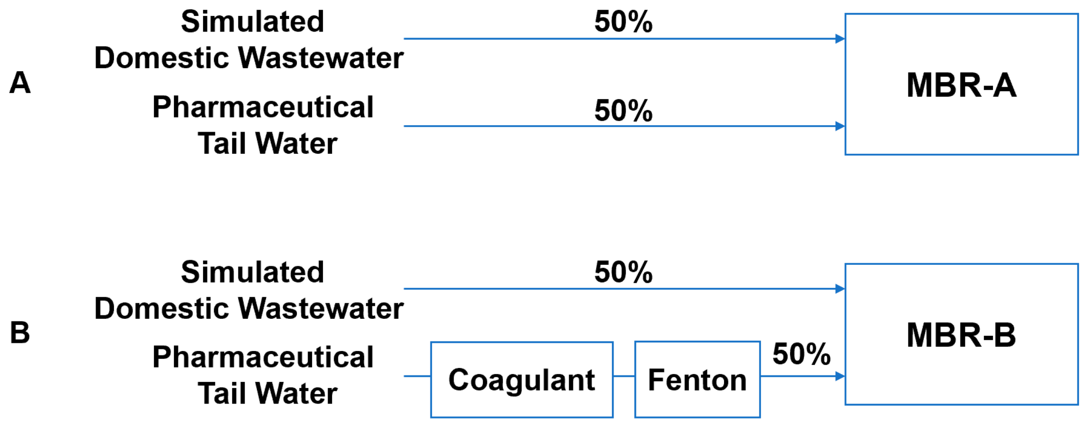

Figure S1 Schematic diagram of the experimental design

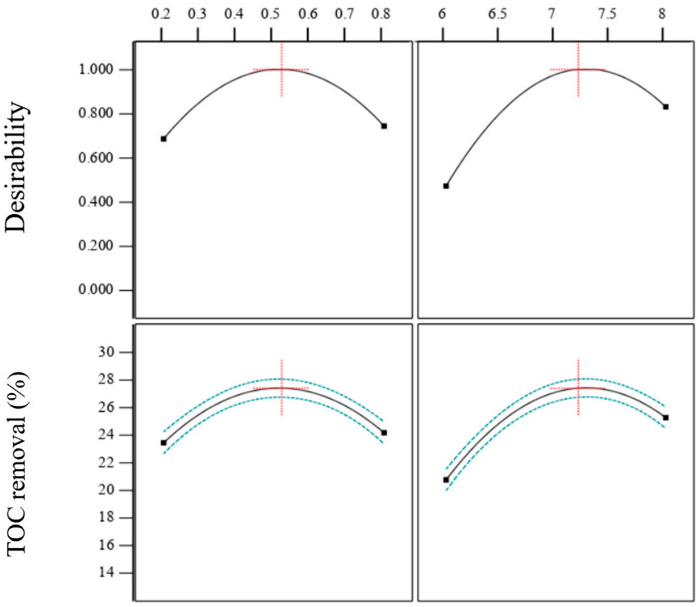

Figure S2 Distribution plot of desirability function values

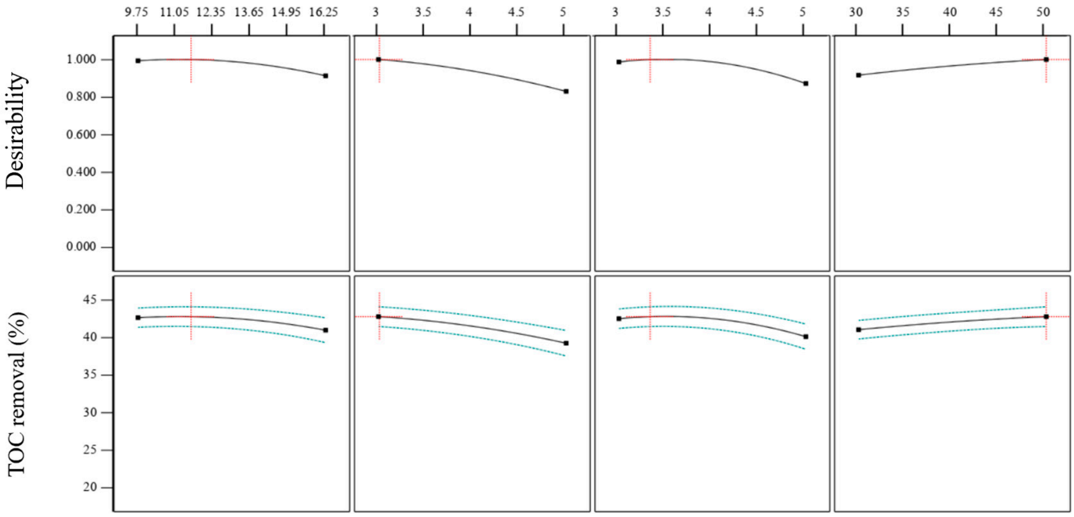

Figure S3 Distribution plot of desirability function values

Table S1 Experimental factor levels

parameters	factors	levels			
		-alpha	-1	1	+alpha
PFS concentration	A	0.0757359	0.2	0.8	0.924264
pH	B	5.58579	6	8	8.41421

Table S2 Design matrix for the two independent variables and the corresponding results at different experimental levels

Trial	PFS concentration (g/L)	pH	TOC removal (%)
1	0.5	7	27.3641
2	0.5	7	27.2135
3	0.5	7	27.3841
4	0.5	7	26.8465
5	0.8	8	20.3235
6	0.2	6	14.8194
7	0.5	5.59	15.9978
8	0.8	6	18.4834
9	0.2	8	22.9922
10	0.5	8.41	22.2946
11	0.5	7	26.5036
12	0.08	7	18.711

Table S3 ANOVA analysis of the TOC removal

Source	Sum of Squares	Mean Square	F value	P value Prob>F	
Model	238.57	47.71	120.21	< 0.0001	Extremely significant
A-PFS concentration	3.62	3.62	9.12	0.0194	Significant
B-pH	44.74	44.74	112.73	< 0.0001	Extremely significant
AB	10.03	10.03	25.26	0.0015	Significant
A^2	81.37	81.37	204.99	< 0.0001	Extremely significant
B^2	116.77	116.77	294.19	< 0.0001	Extremely significant
Lack of Fit	2.20	0.7341	5.10	0.0748	Not significant
Pure Error	0.5762	0.1441			

$p<0.0001$, extremely significant; $p<0.05$, significant.

Table S4 Effect of Different Procedures on Stirring

Program.	Speed 1 of rapid stirring (rpm)	T_1 (min)	Speed 2 of slow stirring (rpm)	T_2 (min)	T_3 (min)	Removal (%)
1	250	2	60	10	60	22.44
2	250	1	60	10	60	21.29
3	250	3	60	10	60	24.46
4	250	2	60	5	60	21.82
5	250	2	60	15	60	22.03
6	250	2	60	10	30	21.09
7	250	2	60	10	120	8.88
8	150	2	60	10	60	14.46
9	350	2	60	10	60	16.74
10	250	2	90	10	60	22.03
11	250	2	30	10	60	21.86

Note: T_1 and T_2 meaning the time of stirrer operated at speed 1 and speed 2, respectively. T_3 indicated the holding time when stirrer was stopped.

Table S5 Experimental factor levels

parameters	factors	levels			
		-alpha	-1	1	+alpha
H_2O_2 concentration	A	3.25	9.75	16.25	22.75
Fe^{2+} concentration	B	1	3	5	7
pH	C	1	3	5	7
Reaction time	D	10	30	50	70

Table S6 Design matrix for the independent variables and the corresponding results at different levels

H_2O_2 dosage (mmol/L)	Fe^{2+} dosage (mmol/L)	pH	Time (min)	TOC removal (%)
16.25	1	1	50	29.89
3.25	1	5	50	40.96
3.25	5	5	10	28.94
9.75	3	3	30	42.51
9.75	3	3	30	40.11
16.25	5	1	10	31.46
9.75	3	3	30	42.19
9.75	3	3	30	42.78
16.25	1	1	10	23.14
9.75	3	3	30	41.16
3.25	1	1	10	21.98
16.25	5	5	50	29.49
3.25	1	5	10	34.17
3.25	5	1	10	31.12
3.25	3	3	30	35.3
3.25	1	1	50	23.32
16.25	5	1	50	37.43
9.75	1	3	30	36.73
3.25	5	1	50	29.78
3.25	5	5	50	31.41
9.75	3	1	30	31.4
16.25	1	5	10	34.13
9.75	7	3	30	32.25
22.75	3	3	30	29.56
9.75	3	3	30	41.87
9.75	3	7	30	25.78
16.25	1	5	50	40.8
9.75	3	3	70	41.19
9.75	3	3	50	42.67
16.25	5	5	10	23.65

Table S7 ANOVA analysis of the TOC removal

Source	Sum of Squares	Mean Square	F value	P value Prob>F	
Model	1252.29	89.45	33.65	< 0.0001	Extremely significant
A-H_2O_2 Concentration	65.28	65.28	24.56	0.0002	Significant
B-Fe^{2+} Concentration	182.74	182.74	68.75	< 0.0001	Extremely significant
C-pH	207.15	207.15	77.93	< 0.0001	Extremely significant
D-Reaction time	28.53	28.53	10.73	0.0051	Significant
AB	2.85	2.85	1.07	0.3171	Not significant
AC	33.44	33.44	12.58	0.0029	Significant
AD	15.94	15.94	6.00	0.0271	Significant
BC	289.26	289.26	108.82	< 0.0001	Extremely significant
BD	4.63	4.63	1.74	0.2065	Not significant
CD	5.12	5.12	1.93	0.1855	Not significant
A^2	141.88	141.88	53.37	< 0.0001	Extremely significant
B^2	60.31	60.31	22.69	0.0003	Significant
C^2	367.14	367.14	138.12	< 0.0001	Extremely significant
D^2	6.93	6.93	2.61	0.1271	Not significant
Lack of Fit	34.99	3.50	3.58	0.0858	Not significant
Pure Error	4.88	0.9764			

p<0.0001, extremely significant; p<0.05, significant.

Table S8 Microorganism diversity index analysis

Sample	Shannon	Simpson	ace	bootstrap	chao	coverage
C	5.3446	0.0206	2357.8206	2507.4095	2306.7249	0.9979
AZ	5.5175	0.0118	1923.4478	1964.9196	1821.9833	0.9945
BZ	5.2285	0.0194	2211.4090	2362.1612	2185.6033	0.9991
MBRAZ	4.8371	0.0263	1574.3461	1603.1660	1499.0593	0.9964
MBR-BZ	5.2882	0.0222	1951.4899	2026.1852	1875.9358	0.9969
